# Travelling for Health

**Published:** 1867-06

**Authors:** 


### HALL'S JOURNAL OF HEALTH.

**Our Legitimate Scope is almost boundless: for whatever begets pleasurable and harmless feelings, promotes Health; and whatever induces disagreeable sensations, engenders Disease.**

WE AIM TO SHOW HOW DISEASE MAY BE AVOIDED, AND THAT IT IS BEST, WHEN SICKNESS COMES, TO TAKE NO MEDICINE WITHOUT CONSULTING A PHYSICIAN.

**Vol. XIV.] JUNE, 1867. [No. 6.**

### TRAVELLING FOR HEALTH.

As many contemplate visiting the old world during the coming summer for purposes of recreation and renovation, our readers will be interested to know from good authority how to do it at least cost and with the greatest advantage to comfort, health and safety.

"In England there is no checking of baggage, and unless you see that your trunks are properly marked and put into the baggage car, you are not at all sure that they will go through; nor even then are you sure of finding them when you arrive at your destination. In France each passenger is allowed fifty-six pounds, but on most of the roads for every ten pounds excess above that you are taxed thirty-five cents. If you have eleven pounds excess you must pay seventy cents. In Italy you must pay for all baggage except that taken in hand. It costs about one-half a fare to take a common-sized trunk through Italy—that is the first cost. Then comes the secondary expenses—every porter expects a fee. A coachman does not descend from his box to lift your trunk—it is not his bnsiness to handle trunks, but a porter is ready at the station door to take it from the coach to the car, for which service he will expect a half-franc. The man who weighs it will ask for a trifle; the clerk who registers it will not give you the baggage-ticket till you have placed a fee in his hand; the man who puts it into the car will politely touch his hat and ask you to remember him. Arriving at your next stopping place, the porter who takes it from the car and carries it to the coach will ask for a half-franc ; the coachman will tell you that baggage is extra and will ask for a trifle that he may drink your honor's health ; the porter at the hotel will make a similar request, and so on at every halting place.

But worse than this leeching of the pocket is the other pother of getting registered at every station. First, you must purchase your passage ticket, then you make your way to the baggage-room to find three or four hundred other persons pushing, crowding, treading on each other's toes—all shouting to the baggage men. It is an unintelligible jargon of Italian, German, French, English, and Spanish. There is always a crowd at one little pigeon-hole where you present your passage ticket, for that must be done before you can have your baggage registered. You are enveloped in an atmosphere of garlic and other nameless and indescribable unsavory smells which arise from the unwashed of Europe.

In many of the stations there is no order or method, and each passenger does what is right in his own eyes, and the strongest and the most adroit is the most successful. Your baggage must be registered ten minutes before the departure of the train, and not unfrequently passengers have the mortification and vexation of seeing a train depart, leaving themselves and baggage behind.

Those who intend making a rapid tour need but little baggage. A gentleman will need only a small carpet-sack. A merchant going from Boston to Chicago, and other Western cities, on business, who intends to be gone six or eight weeks even, does not trouble himself with a trunk—but such a trip is quite as extended as that taken by most European travellers. Distances are short here, when compared with those in America. Thin clothing will not be wanted. One good business suit will suffice for all places, and should any one need new clothing it can be obtained ready made in all the cities and large towns of Europe.

A lady needs a travelling dress of some stout serviceable material—linsey or winsey, proof against mud and water—also one black alpaca, or silk, and perhaps one other dress. Underclothing of every description can be readily obtained, ready made or to order, at cheaper rates than in America, and it is much better to purchase an article when it is needed, than to pay the high transportation that is charged by railway companies. For outward wear, a cloth or black silk sack a breakfast shawl, a blanket shawl, stout, thick-soled walking shoes will give an outfit sufficient for a journey through Europe.

Unless persons tarry long in one place, they do not get into "society," and extra dresses for the drawing-room are not needed. One small trunk will suffice for a gentleman and lady making the tour of Europe, and if Switzerland only is to be visited, two carpet-bags will contain all that will be needed. Most persons who bring large trunks from America leave them in Paris, and travel with the smallest possible amount of luggage.

In Europe few people travel in first-class cars. Men and women high in society, who care to be economical, take the second-class cars of England. The second-class here are about 3½ cents per mile. The first-class is one-third dearer. Hotel bills will be high or low, according to the taste of the individual. Three dollars per day in gold, while travelling, is sufficient to give all necessary comforts. In addition, there are the small fees to those who show you the grand sights, those who have the keys of church doors, the attendants at museums. A thousand dollars in gold will enable a person to see a great deal on this side of the Atlantic, not only the great exhibition, but to take a journey through England, Switzerland, and Germany. Rapid travelling is more expensive than that taken leisurely."

